# Skin cancer margin detection using nanosensitive optical coherence tomography and a comparative study with confocal microscopy

**DOI:** 10.1364/BOE.474334

**Published:** 2022-10-07

**Authors:** Rajib Dey, Sergey Alexandrov, Peter Owens, Jack Kelly, Sine Phelan, Martin Leahy

**Affiliations:** 1 Tissue Optics and Microcirculation Imaging (TOMI) Facility, National Biophotonics and Imaging Platform School of Physics, National University of Ireland, Galway, Galway, Ireland; 2 Center for Microscopy and Imaging, National University of Ireland, Galway, Galway, Ireland; 3 Plastic and Reconstructive Surgery, Galway University Hospital, Galway, Ireland; 4 Department of Anatomic Pathology, Galway University Hospital and Department of Pathology, National University of Ireland, Galway, Galway, Ireland; 5 Institute of Photonic Sciences (ICFO), Barcelona, Spain

## Abstract

Excision biopsy and histology represent the gold standard for morphological investigation of the skin, in particular for cancer diagnostics. Nevertheless, a biopsy may alter the original morphology, usually requires several weeks for results, is non-repeatable on the same site and always requires an iatrogenic trauma. Hence, diagnosis and clinical management of diseases may be substantially improved by new non-invasive imaging techniques. Optical Coherence Tomography (OCT) is a non-invasive depth-resolved optical imaging modality based on low coherence interferometry that enables high-resolution, cross-sectional imaging in biological tissues and it can be used to obtain both structural and functional information. Beyond the resolution limit, it is not possible to detect structural and functional information using conventional OCT. In this paper, we present a recently developed technique, nanosensitive OCT (nsOCT), improved using broadband supercontinuum laser, and demonstrate nanoscale sensitivity to structural changes within *ex vivo* human skin tissue. The extended spectral bandwidth permitted access to a wider distribution of spatial frequencies and improved the dynamic range of the nsOCT. Firstly, we demonstrate numerical and experimental detection of a few nanometers structural difference using the nsOCT method from single B-scan images of phantoms with sub-micron periodic structures, acting like Bragg gratings, along the depth. Secondly, our study shows that nsOCT can distinguish nanoscale structural changes at the skin cancer margin from the healthy region in en face images at clinically relevant depths. Finally, we compare the nsOCT en face image with a high-resolution confocal microscopy image to confirm the structural differences between the healthy and lesional/cancerous regions, allowing the detection of the skin cancer margin.

## 1. Introduction

One of the unresolved clinical issues in skin cancer surgery is the lack of any objectively reliable label-free and accurate assessment modality for resection margin status pre-operatively or intraoperatively. Most commonly, intraoperative visual inspection and palpation are used. This intraoperative technique results in wider surgical margins in almost 40% of the patients [1–3]. Also, this technique is time-consuming. To overcome these clinical challenges, a highly sensitive, non-invasive, cost-effective, and real-time diagnostic tool is required [4]. The use of optical diagnostic technologies such as confocal [5,6], Raman [7], and two-photon [8] microscopy in clinical trials has increased in the past few years. However, most of these techniques are limited to imaging depth up to 100-300 micrometers due to issues with multiple scattering, high numerical aperture, and photon absorption. OCT, in contrast, can image up to a depth of 1 to 2 millimeters in biological samples with high voxel resolution. OCT is a non-invasive optical imaging modality based on low coherence interferometry that enables high-resolution cross-sectional imaging in materials and biologic systems by measuring backscattered light [9]. One major advantage of OCT is that it can be used to obtain both structural and functional information from real-time imaging. Huang *et al*. introduced the principle of OCT in the 1990s [10]. Initially, its clinical use was restricted to the field of ophthalmology [11,12]. Soon after, OCT clinical application extended to several disease diagnoses such as heart and blood vessels [13,14], tumors [15,16] visualization of the dental structure [17,18], diagnosis and treatment of skin disorders [19,20,21,22].

In the last three decades, OCT techniques and tools have improved the resolution to detect micrometer-scale tissue structures. The axial resolution of the conventional OCT system typically depends on the bandwidth and central wavelength of the light source. The lateral resolution depends on the numerical aperture of the sample arm objective lens [23]. Over the years, to improve the axial resolution, supercontinuum laser sources have been used [24]. In general, conventional OCT commonly has non-isometric resolutions, particularly with poorer lateral resolutions on the order of 10 µm or greater. Objective lenses with high numerical aperture are used in conventional OCT systems to achieve near-isometric resolution and detect fine structural features within a limited imaging depth of a volume object [25,26,27]. Sensitivity and scanning speed are two other major parameters of the OCT system. The scanning speed and sensitivity significantly improved by Fourier domain OCT (FDOCT) including spectral-domain OCT (SDOCT) and swept-source OCT (SSOCT) in comparison with time-domain OCT (TDOCT) [28].

Recently Bernstein *et al.* demonstrated a supercontinuum-based ultrahigh-resolution spectral-domain OCT system with a central wavelength of 1300 nm and axial resolution of 2.6 µm in the air [29]. However, the resolution limits persist, and sub-micron structural features remain inaccessible to OCT. Structures smaller than approximately half of the illumination wavelength were not resolvable. However, structures smaller than the diffraction limit still affect the scattering events. This emerges as granular artifacts in the OCT image known as speckle. The fundamental idea of speckle is that random interference within a resolution voxel occurs, so that a dark pixel in an image does not always represent a weaker scattering structure. Recently, a few OCT methods to detect sub-micrometer structural changes within biological tissue have been described. Inverse spectroscopic OCT (ISOCT) was introduced by measuring the wavelength-dependent scattering and backscattering coefficient to quantify the mass-density function. They show that the length scale of sensitivity of ISOCT ranges from 30 to 450 nm without resolving specific geometrical features [30,31]. Another technique is based on the phase-sensitive OCT (PSOCT) to characterize the motion of cellular components within the organ of Corti at a sub-micron scale [32]. Nanosensitive OCT (nsOCT) is a novel imaging technique based on the spectral encoding of spatial frequencies (SESF) principle which was used for real-time and super-resolution imaging [33–38]. The nsOCT technique visualizes sub-wavelength structure without resolving it and provides nanoscale sensitivity to structural changes from single frame [39–44,46]. An advantage of this technique has been its use for real-time and super-resolution imaging [37,38]. Each local frequency component of the scattering object is encoded in a corresponding optical illumination wavelength range. Recently, nsOCT has been applied to detect both structural [42–44] and functional [45] changes in *ex vivo* and *in vivo* biological tissues including visualization of the nanoscale structural changes within mesenchymal stem cells (MSC) [46]. The nsOCT gives the information content of the FDOCT signal which is based on the general scattering theory. In our previous publication [39–44,46], we showed that the FDOCT signal in the axial component of the 3D Fourier transform of spatial frequencies is limited by illumination bandwidth. We also explained the reason why high spatial frequency information is absent in conventional OCT image. In addition, we demonstrated how this high spatial frequency information can be mapped to each volume of interest within the FDOCT image. Therefore, besides detection of the alteration of the structure smaller than spatial resolution, nsOCT approach permits visualization of the dominant spatial period of the sub-wavelength structure at each small volume of interest within the FDOCT image. Despite some similarities in processing between spectroscopic OCT (SOCT) [47–49] and nsOCT, the nsOCT technique is fundamentally distinct from SOCT, which is often based on sample absorption properties or on models such as Mie scattering theory to compute the size of spherical scatterers. The interpretation of the nsOCT and SOCT results also differ. To our knowledge, there is no publication where the spectroscopic OCT was used to reconstruct the local Fourier spectra of spatial frequencies within the sample.

In the last decade, OCT in the field of Dermatology has been less established than in Ophthalmology since the scattering of light strongly limits the penetration into the skin [20]. Nowadays, OCT has been increasingly used to diagnose, monitor treatment response, and plan excisions of skin cancers and non-cancerous skin diseases [50]. Recently, line-field confocal optical coherence tomography (LCOCT) combined the benefits of high penetration depth of OCT and high-resolution, equivalent to reflectance confocal microscopy to acquire the volume image from the diseased skin [51]. However, this technique cannot give nanometer length scale sensitivity from the tissue structure’s volume image. The most prevalent nonmelanoma skin cancer among Caucasians is basal cell carcinoma (BCC) [52]. The second most common form of skin cancer is squamous cell carcinoma (SCC). Both BCC and SCC arise in the basal layer of the epidermis. It is well known that many cancers have their first manifestations at the nanoscale level, and these nanoscale structural changes start at the cellular level before appearing at the tissue level. Non-invasive nsOCT has great potential to detect sub-cellular nanoscale structural features at clinically relevant depths.

In the present study, we investigate the usefulness of the nsOCT technique, using a newly developed SDOCT system with a broadband supercontinuum light source, in three different ways. The wider spectral bandwidth allows access to a larger range of spatial frequencies and improves the dynamic range of the nsOCT. Firstly, we numerically validate the nsOCT approach on synthetic submicron axial structures made to a few nanometers tolerance. Secondly, we experimentally validate the nsOCT approach from two periodic Bragg grating samples with known dimensional sizes. We apply the nsOCT method in assessing and discriminating between *ex vivo* healthy skin and cancer margin of the resected surgical specimens. Finally, we compare nsOCT image information with high-resolution confocal microscopy images.

## 2. Materials and methods

### 2.1 High-resolution broadband spectral-domain OCT system

In this study, for spectral interference data collection, a custom built fiber-based SDOCT system is used with supercontinuum laser light and a broadband spectrometer. The SDOCT system has an axial and lateral resolution of 3.8 µm in air and 5.5 µm, respectively [53]. This SDOCT system operates within a wavelength range of 1100 nm to 1500 nm (400 nm bandwidth) with a central wavelength of 1300 nm. The system has a sensitivity of 109 dB at 73 kHz line rate. Detectable spatial period or spatial frequency range depends on the light source bandwidth and spectrometer bandwidth range. The data acquisition process was performed through our home-built software platform written in LabVIEW. All post-processing steps were done separately in MATLAB after data acquisition to reconstruct conventional B-scan images and nanosensitive OCT (nsOCT) images. In the previous studies [42,42,46] we used commercial SDOCT system (Telesto III, Thorlabs.inc) with centered at 1300 nm (bandwidth 237 nm, wavelength range 1176-1413 nm).

### 2.2 Nanosensitive optical coherence tomography

Nanosensitive OCT is a post-processing computational form of OCT that detects the spatial and temporal structural changes within the three-dimensional scattering objects. The nsOCT method is based on the theory of spectral encoding of spatial frequency (SESF) approach, which allows detection of structural changes of the objects with nanoscale sensitivity [36–40].

To construct the nsOCT images, first we processed the interference spectra after basic preliminary signal processing, including background noise removal, apodization, *k*-space linearization, and dispersion compensation. OCT is typically a one-dimensional solution of the inverse scattering problem. The collected axial spatial frequency can be expressed as

(1) $$\nu_{z}=n(cos\theta +cos\alpha )/\lambda$$

where *θ* and *α* are the illumination and scattering angles. *n* and *λ* are the refractive index of the medium and illumination wavelength. For the reflection configuration at small illumination and scattering angles *θ* and *α* are ≈ 0, which is typical for OCT [49]. Then the processed interference spectra are rescaled from wavelength to frequency. After that, the collected complex amplitudes of the spectra are converted to complex amplitudes of spatial frequency based on the following relation, which is a simplified form of Eq (1).

(2) $$\nu_{z}=\frac{2n}{\lambda }$$

where *n* is the refractive index, and *λ* is the wavelength of the light source [37]. The spatial frequency range is subsequently calculated as

(3) $$\Delta \nu_{z}=2n\Delta \lambda /(\lambda_{1}\lambda_{n})$$

Where Δ*λ* = *λ_n_* - *λ*_1_ is the light source bandwidth, *λ_n_* and *λ*_1_ are the longest and shortest wavelengths of the light source spectrum.

The rescaled spectra of the complex amplitude of spatial frequencies are decomposed into the sub-bands, for example, using the Tukey window. The IFFT of each sub-band signal has been performed to get the amplitude along all the imaging depths for one spatial frequency range. Finally, by combining all the sub-band spatial profiles, it is possible to reconstruct the axial spatial frequency profile at each image pixel/voxel. After that the dominant spatial frequency/period can be calculated from each voxel. The relation between the spatial periods of the structures and the spatial frequencies is as follows

(4) $$H_{z}=1/\nu_{z}$$

From the dominant spatial period of each voxel, an nsOCT image can be formed using an optimized threshold [37]. In the present study, Δ*λ* is 400 nm where the corresponding *λ*_1_ to *λ_n_* range is 1100 to 1500 nm. According to Eq. (4) physical spatial periods varied from 392.8 nm to 535.7 nm (after correction using *n* = 1.36 for human skin). For analysis, we used physical spatial periods within the length scale of sensitivity.

### 2.3 Submicron axial periodic structures and numerical simulation to construct nsOCT image

The nsOCT technique was used to image two samples with sub-micron periodic structure along depth (reflection Bragg gratings) from OptiGrate Corp. USA, as proof of concept. These two samples have different axial periodic structures, and the periodicity is 431.56 nm and 441.76 nm. Each periodic layer was fabricated using a sinusoidal refractive index variation of 1.483 ± 0.001. The periodicity of the sample remains stable for ambient temperatures up to 400°C. Each sample has a thickness of ∼3 mm. To mimic this experimental condition, we synthesized and performed numerical simulation using three different axial periodic structures with periodicities of 431.56 nm, 441.76 nm, and 445.04 nm. In these axial periodic structure simulations, we followed the same sinusoidal refractive index variation as Bragg grating samples. In our previous paper, we showed the simulation of randomized submicron axial structures with different periodicities at different depths to mimic the tissue-like structure [47]. We demonstrated that a few nanometer structural differences can be detected between the randomized axial structures along the depth direction. In our present simulation strategy, we applied this to an OCT system with a broadband supercontinuum laser and followed standard SDOCT image construction theory to implement the proposed nsOCT simulation [54,55]. To mimic the same fabricated layer sample, in the simulation, we divided each periodic layer into many sublayers with a thickness of 0.01 nm to make it smooth enough for sinusoidal refractive index variation. For example, one sample has 431.56 nm periodicity and the number of sublayers in each periodic layer is 43,156. The SDOCT interference signal was formed from the different layers of the synthesized structure and the reference wave of the gold-coated mirror reflection. In the simulation, we added some background noise from the detector spectra to mimic the same experimental condition. To match the experimental conditions, we used a signal-to-noise ratio of ∼ 69 dB in the simulation. Finally, the B-scan image was formed after the inverse Fourier transform of the interference signal. In OCT simulation the same sinusoidal refractive index variation was considered for all three samples with layer thicknesses of 431.56 nm, 441.76 nm, and 445.04 nm. From these simulations, we have demonstrated that our state of art nsOCT method can detect depth-resolved submicron structures with an accuracy of a few nanometers.

### 2.4 Tissue sample preparation

This research study was approved by the Galway Clinical Research Ethics Committee and the approved reference number is *C.A. 2575*. All the fresh tissue samples from human skin are prepared in accordance with the guidelines of the Department of Anatomic Pathology, Galway University Hospital. Tissue samples are examined by a pathologist, who documents any gross abnormalities and applies ink to the margins of the specimen, prior to sectioning the specimen into 2 mm slices to be processed for microtomy and H&E staining to facilitate pathological diagnosis. Concomitantly a representative section of tissue, including tumor and adjacent normal tissue is taken for this research study. All the tissue samples were kept into 4% paraformaldehyde (PFA) solution to keep the samples fresh and to minimize the structural changes. Only during the OCT imaging, all the tissue samples were removed from the PFA solution for a minimum period to avoid dehydration. After finishing the OCT imaging from the same tissue sample, one slice was taken for histology imaging and the rest of the tissue block was fixed using paraffin for high-resolution confocal microscopy imaging with the help of pathologists. During histology and confocal microscopy imaging, tissue samples were kept in the same orientation as the OCT image.

## 3. Results and discussion

**Fig. 1.**
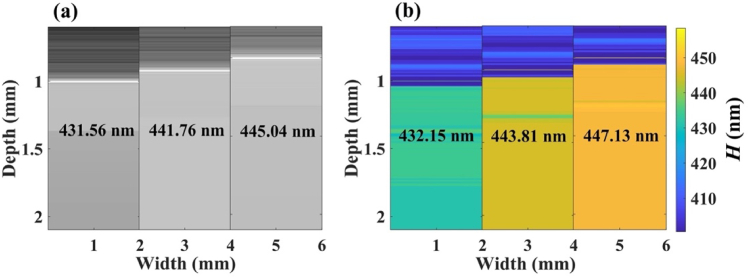
(a) Simulated OCT B-scan images from three different periodic structures and corresponding original spatial periods. (b) nsOCT B-scan images from (a) along with the detected submicron structural sizes.

To validate the nsOCT technique, we constructed intensity-based OCT B-scan images from three different synthetic axial structures. In Fig. 1(a), we show the OCT B-scans from the synthetic structures along with their axial structural size. Figure 1(b) shows numerically constructed dominant spatial periods mapping nsOCT image from the Fig. 1(a) OCT B-scan image. Detected submicron axial structural sizes are mentioned on each nsOCT image. From this numerical nsOCT technique, it is clearly seen that the submicron axial structure is detectable within the ∼2 nm accuracy.

**Fig. 2.**
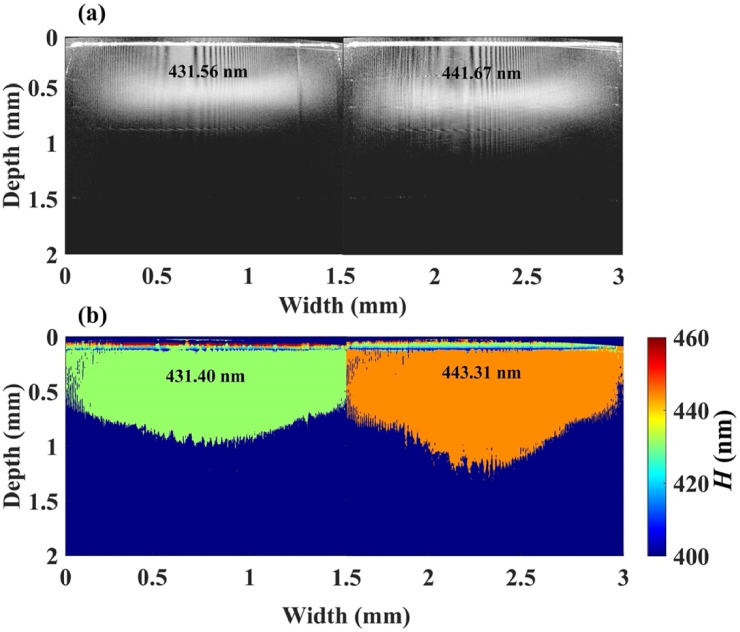
Experimental demonstration of nsOCT technique using Bragg gratings, the detected axial spatial periods are 431.56 nm and 441.67 nm. (a) OCT B-scan image, (b) nsOCT B-scan image of the two Bragg gratings. The color bar represents structural size in nanometers.

To demonstrate the potential of nsOCT technique to detect axial submicron structures experimentally, we imaged two Bragg gratings from OptiGrate Corp, USA with periodicities of 431.56 nm and 441.76 nm, respectively. Figure 2(a) represents the conventional intensity-based OCT B-scan image from the two periodic samples. Figure 2(b) shows the corresponding nsOCT B-scan images formed as a color map from the detected spatial periods. The information from high spatial frequency content is not preserved in conventional OCT processing. Consequently, the structural difference between the two samples cannot be detected in conventional intensity-based OCT B-scan images. However, from the proposed nsOCT method ∼10 nm axial structural difference is easily visualized between the two Bragg gratings with ∼2 nm detection accuracy. Each Bragg grating has a ∼150 nm antireflection coating on the top surface which can be seen with a different color in Fig. 2(b) nsOCT B-scan image. Figure 2(b) nsOCT B-scan image confirms that using the nsOCT method, we can detect nanoscale structural alterations within submicron axial structures.

**Fig. 3.**
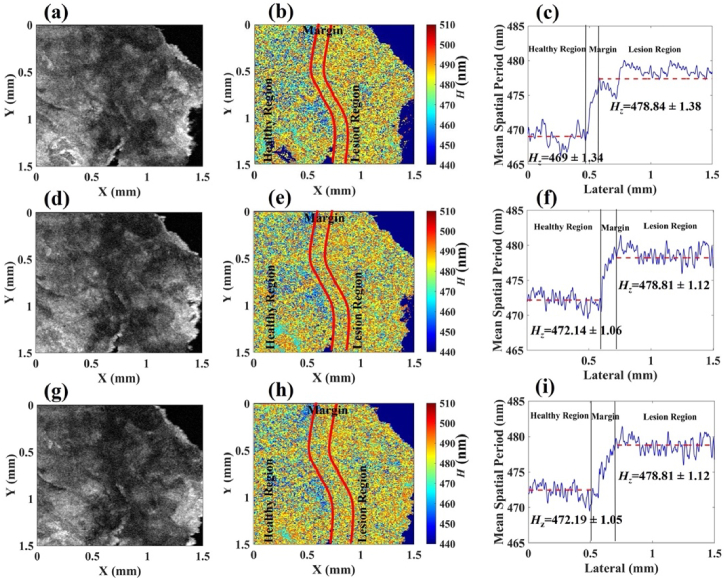
(a), (d), and (g) conventional intensity-based OCT en face images of the tissue at the depths of ∼200 µm, 400 µm, and 600 µm respectively. (b), (e), and (h) are corresponding nsOCT en face images with spatial period mapping, presenting the dominant structural size. The healthy, marginal, and lesional regions are separated by the red lines. The color bar of the nsOCT en face images represents the spatial periods in nanometers. Plot (c), (f), and (i) show changes in the mean dominant spatial period of the structure between the two regions, defining an intervening marginal area in the nsOCT en face images.

After successful numerical and experimental validation of the nsOCT approach, we applied the nsOCT technique to fresh human skin cancer tissue samples. We collected skin tissue samples with healthy and lesional parts within one sample. After 3D OCT image acquisition from those collected tissue samples, the nsOCT technique was applied to distinguish both regions with an intervening margin area. Here, we show three conventional OCT and corresponding nsOCT en face images from 3D volume image (we have added results from two more tissue samples in the supplementary material to underline the capacity of the nsOCT technique to detect the margin between the healthy and lesional regions). Figures 3(a), 3(d), and 3(g) represent conventional intensity-based OCT en face images at depths of 200 µm, 400 µm, and 600 µm (from the top surface of the tissue), respectively from the acquired 3D volume. It is impossible to detect structural changes between healthy and lesion regions from the conventional OCT en face images in Fig. 3(a), 3(d), and 3(g). Figures 3(b), 3(e), and 3(h) represent nsOCT en face images which were constructed from the complex spectral interference OCT signal. In the nsOCT en face images, assorted color code mapping represents the different dominant nanoscale structural sizes of the tissue. Within the nsOCT en face images, a null value for accurate spatial period measurement is set as a dark blue background color and does not represent any structure. In contrast to conventional OCT, the nsOCT image is formed using different contrast mechanisms like the dominant size of the sub-micron structure at a given location. Figures 3(c), 3(f), and 3(i) show the change in mean spatial periods from the healthy region to the lesion region of the nsOCT en face images 3(b), 3(e), and 3(h) respectively. The mean spatial period was calculated from the nsOCT en face images. From Fig. 3(c), it can be clearly observed that from the healthy region to lesional region the mean spatial period (*H_z_*) value increased from 469 ± 1.34 (mean spatial period ± standard deviation) nm to 478.84 ± 1.38 nm. Similarly, Fig. 3(f) and 3(i) shows the mean spatial period changing from 472.14 ± 1.06 nm to 478.81 ± 1.12 nm and from 472.19 ± 1.05 nm to 478.81 ± 1.12 nm, respectively. Red dashed lines in Fig. 3(c), 3(f), and 3(i) represent the mean spatial period values of each region. So, the mean spatial period of the structure has been increased from left to right side within the nsOCT enface images. In the nsOCT en face images, the right side of the image represents higher periodicity, and the left side of the image represents lower periodicity. A higher spatial period or larger structural size represents the lesion region and a lower spatial period or smaller structural size represents the healthy region of the tissue. According to the mean spatial period distribution in Fig. 3(c), 3(f), and 3(i) the region where the slope changes represent the margin between the healthy and lesional regions of the tissue. The width of the margin is represented by the spacing between the two black straight lines in the Fig. 3(c), 3(f), and 3(i). In the nsOCT enface images, the spacing between the two red curved lines represents the margin between the healthy and lesional regions. The spacing between the red curves in Fig. 3(b), 3(e), and 3(h) is determined according to the spacing between the two black straight lines in Fig. 3(c), 3(f) and 3(i). From Fig. 3(a), 3(d), and 3(g) it can be observed that conventional intensity-based OCT en face images cannot distinguish healthy and lesional regions. It can give only the intensity value at each point, which provides only reflectivity at a given location and does not convey any information about the structures below the resolution limit at that location. In contrast, the processed nsOCT en face images and the mean spatial period profile of these nsOCT en face images clearly show the differences in the spatial period within the healthy and lesional regions. Similar to these, nsOCT en face images and mean spatial period profiles from two other tissue examples are presented in Figs. S1(b)-(c) and S1(e)-(f) in the supplemental material.

**Fig. 4.**
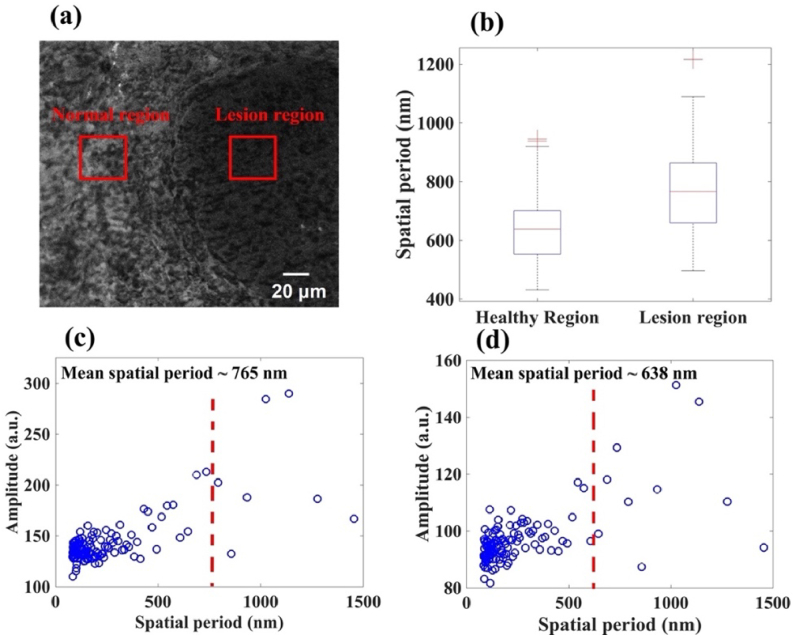
(a) Confocal microscopy image of the tissue sample. Line profiles of the selected red square box regions were taken for spatial period profile measurements using Fourier transform (b) box plot showing the spatial period distribution of the healthy and lesional regions. (c) and (d) spatial period profile obtained from selected lesional and healthy regions of the tissue.

Currently, stained tissue slides of formalin-fixed or paraffin-embedded tissue section images using a bright-field microscope are the gold standard to evaluate micron scale morphological features. In addition to the nsOCT results to analysis the nanoscale structural changes, laser scanning confocal microscopy images were used for structural comparison. After OCT imaging, the same tissue sample was fixed with paraffin for confocal microscopy imaging. Laser scanning confocal microscopy enables imaging of lesional and healthy skin tissue at cellular resolution in the freshly excised fixed tissue block [5]. We used a 405 nm excitation wavelength and an objective lens with an *NA* (Numerical Aperture) of 0.9 for acquiring the image. The image consisted of 4096 × 4096 pixels covering an area of 225 × 225 µm, which corresponds to 55 nm/pixel with a lateral resolution of 166 nm. The confocal microscopy image of the tissue block is presented in Fig. 4(a). To validate the nsOCT results with the confocal microscopy image, spatial frequency changes of the structures within the two regions were calculated. To analyze the spatial frequency profiles, regions of interest marked by red square boxes were taken from the confocal image 4(a). We have analyzed two hundred vertical line profiles from the red square boxes. To obtain the spatial period distribution of the structures, we applied Fourier transforms on these line profiles. The median spatial frequency/period (MSP) of the spectrum was calculated after the Fourier analysis of the spectrum profiles [47]. From the two hundred vertical line profiles, average MSP was calculated (according to equation S1 in the supplemental material) to distinguish the spatial period changes of the structures within the healthy and lesional regions. Figures 4(c) and (d) show the spatial period profile of the median spatial period values from the lesional and healthy regions, respectively. In Fig. 4(c) and (d) the red dotted line represents the median spatial period values from the lesional and healthy regions. From the red dotted line, it can be observed that the median spatial period is higher, or it is shifted towards the right of the spectrum for the lesional region. It indicates an increase in the spatial period of the structures within the lesional region compared to the healthy region. Figure 4(b) box plot shows the distribution of the mean spatial periods of healthy and lesional regions and it is calculated from the confocal image line profile of Fig. 4(a). The box plot indicates that the spatial period of the nanoscale structures within the lesional region increased compared to the healthy region. The mean spatial period distribution of the healthy region is 638 ± 107 nm (mean SP ± standard deviation) and for the lesional region is 765 ± 135 nm. From the confocal microscope image, each red boxes line profile was analyzed for statistical t-test comparison of the median spatial period values of the healthy and lesional regions. Paired t-test results show a significant difference (p < 3.48 × 10^−24^) in spatial period between the healthy and lesional regions. Thus, the Fourier analysis results of the confocal microscopy images support the results acquired using the nsOCT technique. The higher structural size in the lesional region could be attributed to nuclear enlargement, increased nuclear-to cytoplasmic ratio, nuclear membrane irregularities, hyperchromasia, and abnormal chromatin distribution. In the lesional region, cells are also likely to have an increased volume of genetic material as well as altered nuclear envelope shape. All these factors may also contribute to the differing observation between healthy and lesional regions [56]. Particularly in skin, the basal layer of the epidermis contains multiplying keratinocytes. Terminally differentiating progeny leave this layer and enlarge progressively as they move outward through the spinous layer. The size of the cell is related to the degree of its terminal differentiation. In cell culture studies, human keratinocyte nuclei were found to vary from 3-10 microns to 11-20 microns in diameter [57–59] during progression from benign to malignant. Further investigation is required to compare the nsOCT results with the nuclear components structural sizes of the cancer cell.

## 4. Conclusions

In this study, we have shown that differences between the nanoscale structure of healthy and lesional/cancerous regions, undetectable using conventional OCT, can be clearly detected by the nsOCT approach. Several experiments from different tissue samples have consistently shown larger dominant spatial periods in the lesional region compared to the healthy region. Using nsOCT we demonstrated the ability to detect the depth-resolved structural changes within a 3D tissue sample and visualize the margin between the healthy and lesional/cancerous regions at different depths. The detected dynamic range of the nanometer length scale sensitivity of the tissue structure depends on the illumination source spectral bandwidth. All the detected tissue structural sizes are within our illumination bandwidth range. There may be some other axial structural sizes within the tissue beyond our detection range. To detect those axial structures, further investigation with a larger illumination source bandwidth OCT system would be required. As a comparative study, confocal microscopy was used to detect structural changes at different bandwidths of structural sizes, with higher spatial periods, and this image shows a similar trend to the nsOCT results. A corresponding study with other bandwidths of the lower spatial frequencies was done using histological images, Fig. **S3**. The results demonstrate that for both nsOCT and confocal microscopy, the measured spatial period, or dominant structural sizes in the lesional region is higher compared to the healthy region for both images.

We believe that the nsOCT method can be used as a tool to detect and monitor skin cancer at an early stage, possibly in a pre-invasive phase, which may facilitate early clinical intervention. Implementation in real-time would allow more confident surgery and avoid hospital stay/revisit. Morphologically, the cancerous cell is characterized by a larger irregular nuclear size, occasionally multiple. Our nsOCT and confocal microscopy results support the visible microscopic changes in the cell nuclei that are well established using the current gold standard histology method. All the tissue samples used were collected post-operatively, following pathological confirmation of the histological diagnosis. To further develop this technique, *in vivo* imaging during the biopsy process and comparison with the nsOCT technique to detect the healthy and lesional/cancerous region along with the marginal area is planned. Further, the nsOCT technique can be used to study the morphological changes of cancerous cells compared to normal cells, as they undergo the nanoscale structural changes within their progress and long before they manifest as a disease.

## Data Availability

The data supporting this study’s findings are available from the corresponding author upon reasonable request.

## Supplemental document

See Supplement 1 for supporting content.
