# Supplementary material for: Skin cancer margin detection using nanosensitive optical coherence tomography and a comparative study with confocal microscopy
Source: Biomed Opt Express. 2022 Oct 7;13(11):5654–66. doi: 10.1364/BOE.474334 (PMC9872867; doi:10.1364/BOE.474334)
Supplement: Supplementary file 1 [file boe-13-11-5654-s001.pdf]

Supplemental Document

Biomedical Optics EXPRESS

Skin cancer margin detection using nanosensitive optical coherence tomography and a comparative study with confocal microscopy: supplement

RAJIB DEY,[1] SERGEY ALEXANDROV,[1] PETER OWENS,[2] JACK KELLY,[3] SINE PHELAN,[4] AND MARTIN LEAHY[1,5,*]

[1] *Tissue Optics and Microcirculation Imaging (TOMI) Facility, National Biophotonics and Imaging Platform School of Physics, National University of Ireland, Galway, Galway, Ireland*
[2] *Center for Microscopy and Imaging, National University of Ireland, Galway, Galway, Ireland*
[3] *Plastic and Reconstructive Surgery, Galway University Hospital, Galway, Ireland*
[4] *Department of Anatomic Pathology, Galway University Hospital and Department of Pathology, National University of Ireland, Galway, Galway, Ireland*
[5] *Institute of Photonic Sciences (ICFO), Barcelona, Spain*
[*] *martin.leahy@nuigalway.ie*

This supplement published with Optica Publishing Group on 7 October 2022 by The Authors under the terms of the Creative Commons Attribution 4.0 License in the format provided by the authors and unedited. Further distribution of this work must maintain attribution to the author(s) and the published article's title, journal citation, and DOI.

Supplement DOI: https://doi.org/10.6084/m9.figshare.21268479

Parent Article DOI: https://doi.org/10.1364/BOE.474334

Skin cancer margin detection using nanosensitive optical coherence tomography and a comparative study with confocal microscopy

RAJIB DEY[1], SERGEY ALEXANDROV[1], PETER OWENS[2], JACK KELLY[3], SINE PHELAN[4], AND MARTIN LEAHY[1,5*]

[1]*Tissue Optics and Microcirculation Imaging (TOMI) Facility, National Biophotonics and Imaging Platform School of Physics, University of Galway, Galway, Ireland*

[2]*Center for Microscopy and Imaging, University of Galway, Galway, Ireland*

[3]*Plastic and reconstructive surgery, Galway University Hospital, Galway, Ireland*

[4]*Department of Anatomic pathology, Galway University Hospital and Department of Pathology, University of Galway, Galway, Ireland*

[5]*Institute of Photonic Sciences (ICFO), Barcelona, Spain*

**martin.leahy@nuigalway.ie*

Other tissue sample nsOCT results

We show another two samples of nsOCT results for further structural comparison of the healthy and lesional regions with an intervening marginal area. These two samples were collected from the nose and scalp of human skin. Fig. (a) and (d) represent conventional intensity-based OCT en face images from the acquired volumes. Fig. (b) and (e) represent the nsOCT en face images. Fig. (c) and (f) show the changing of mean spatial periods from the healthy region to the lesion region of the nsOCT en face images (b) and (e). From Fig. (c), it can be clearly observed that the lesional region to healthy region mean spatial period (H_z) value changed from 482 ± 1.28 (mean SP ± standard deviation) nm to 475.68 ± 1.37 nm. Corresponding Fig (f) show the changing of the mean spatial period from 473.68 ± 1.01 nm to 479.71 ± 0.98 nm. Red dashed lines in figs. (c) and (f) represent the mean spatial period values of each region. As a result, the mean spatial period of the structures has increased from the healthy region to the lesional region. All three samples followed the similar mean spatial period profiles trained.

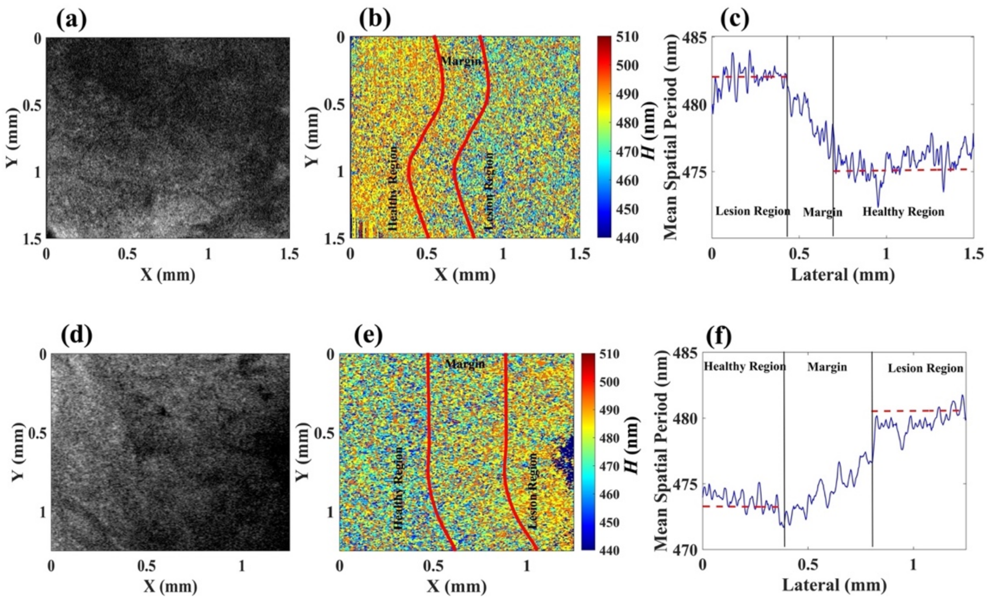

Fig. S1. (a) and (d) conventional intensity-based OCT enface images from the nose and scalp. (b) and (e) the corresponding nsOCT en face images with spatial period mapping, presenting the nanoscale structural alterations. At the depth of ~423 mm and ~455 mm the en face images were constructed. The color bar of the nsOCT en face images represents the spatial periods in nanometers. (c) and (f) show changes of the mean dominant spatial period between the two regions with an intervening marginal area of the nsOCT en face images (b) and (e).

Tissue sample overview from OCT en face image, confocal image, and H&E slide image

To get an overview of the tissue sample, parallelly we have shown three different system images with the same sample orientation. Fig. (a) represents a conventional intensity-based OCT en face image. As shown in Fig (a), intensity-based OCT en face image cannot discriminate between healthy and lesion regions. Fig. (b) represents a laser scanning confocal microscopy image of the same tissue block. The right side of the image appears to lesion region and the left side is the healthy region. 20X objective lens with an 0.75 NA (Numerical Aperture) has been used for confocal microscopy imaging. Fig. (c) represents a low-resolution H&E slide image to get an overview of all the regions of the same tissue. To acquire this H&E slide image, a 4X objective lens with 0.13 NA has been used to image healthy and lesion regions.

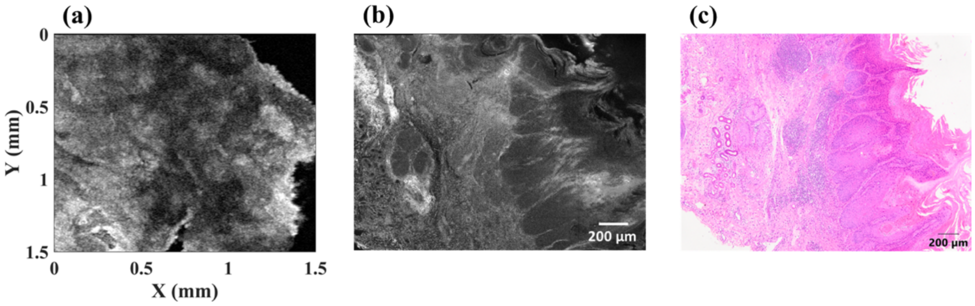

Fig. S2. (a) Conventional intensity-based OCT en face image of the tissue. (b) Laser scanning confocal image of the tissue sample. (c) H&E slide image of the tissue sample.

Histology image analysis results

We used an Olympus light microscopy of 580 nm excitation wavelength and an objective lens with an NA (Numerical Aperture) 0.75 for acquiring the H&E slide image. Image consisted of 2056 X 1920 pixels covering an area of 283 X 212 mm, which corresponds to 110 nm/pixel with a lateral resolution of 286 nm. The light microscopy image of the H&E slide is presented in Fig. 4(a). To validate the nsOCT results with the light microscopy image, spatial frequency changes of the structures within the two regions have been calculated. To analyze the spatial frequency profiles black square boxes regions were taken of the light microscope image 4(a). We have analyzed two hundred vertical line profiles from the black square boxes. To obtain the spatial period distribution of the structures, Fourier transforms have been applied on these line profiles. Median spatial frequency/period (MSP) of the spectrum was calculated after the Fourier analysis of the spectrum profiles. Figure 4(b) box plot shows the distribution of the mean spatial periods of healthy and lesion regions, and it is calculated from the line profiles of fig 4(a). The box plot indicates that the spatial period of the nanoscale structures within the lesional region increased compared to the healthy region. The mean spatial period distribution of the healthy region is 837 ± 123 nm (mean SP ± standard deviation) and for the lesional region is 1000 ± 151 nm.

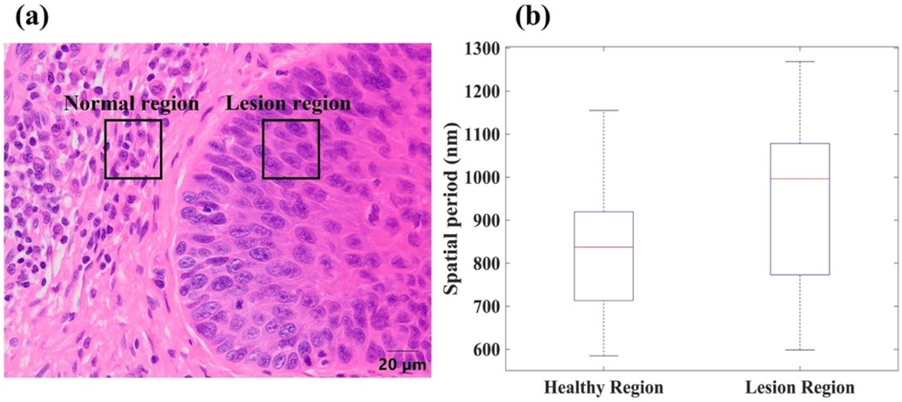

Fig. S3. (a) Histological sectioning image of the tissue sample. Line profiles of the selected black square boxes region were taken for spatial period profile measurements using Fourier transform (b) box plot showing the spatial period distribution of the healthy and lesional regions.

Confocal and histology image analysis flowchart

In order to analyse the spatial frequency profiles from the confocal and H&E slide images, we analysed two hundreds vertical line profiles across the confocal image in a given region and Fourier transform of these profiles were calculated to obtain the spectrum of spatial period distribution of the structures as shown in flowchart below. The regions within the confocal image were carefully chosen to avoid any artefacts caused by fixation/folding. From the Fourier spectrum of the profiles, median spatial frequencies/periods of the spectrum were calculated using Eq. S1.

$$msf = \frac{\sum_{n=1}^{N} f(n)\, x(n)}{\sum_{n=1}^{N} x(n)} \qquad \text{(S1)}$$

where *x(n)* represents the intensity of the spatial frequency/period profile at the frequency/period *f(n)*. This method characterizes the median local structure size within the sample.

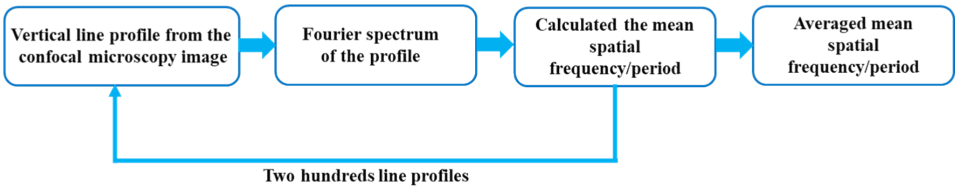

nsOCT image formation

In order to realize nsOCT methods, we first obtain the processed interference spectra after basic preliminary modifications, including k-space linearization, background noise removal, apodization, and dispersion compensation. Then the k-space linearized spectral interferogram $I(\lambda)$ is converted to corresponding axial spatial frequency $I(v_z)$ using Eq. (2). Next the processed spectra of axial spatial frequency are decomposed into several sub-bands (N=10 number of sub-bands) using Tukey windows as for nsOCT imaging. We can use different window widths based on the sample and imaging aim to improve structural or spatial resolution. For each of the N zones, the axial spatial frequency profile is inverse Fourier transformed to reconstruct the OCT image for each zone. Zero-padding was used before FFT for each zone to better localize the peak frequency. From the reconstructed OCT images of N zones for each point of depth profiles we form an energy contribution versus the corresponding spatial period of the sub-band plot, called the spatial frequency/period profile. Then, the nsOCT image can be formed as a color map of some informative parameters of these spatial frequency profiles which better visualize the structural changes. In this paper the dominant spatial frequency / period value which corresponds to maximum energy contribution is mapped to create the nsOCT image. This procedure is repeated for each A-line in a B-scan, as well as for each B-scan in a 3D volume. After the Fourier transformed from each zone, we subtracted the noise using thresholding. Furthermore, a 4 x 4 spatial kernel and a threshold value of 1.8 was applied to suppress noise within the nsOCT images for this application. Depending on the application or purpose, the spatial filtering kernel and the threshold can be optimized.
